# Drug repurposing and computational modeling for discovery of inhibitors of the main protease (M^pro^) of SARS-CoV-2†

**DOI:** 10.1039/d1ra03956c

**Published:** 2021-07-02

**Authors:** José Rogério A. Silva, Hendrik G. Kruger, Fábio A. Molfetta

**Affiliations:** Laboratório de Planejamento e Desenvolvimento de Fármacos, Instituto de Ciências Exatas e Naturais, Universidade Federal do Pará Belém Pará 66075-110 Brazil rogerio@ufpa.br; Catalysis and Peptide Research Unit, University of KwaZulu-Natal Durban 4000 South Africa; Laboratório de Modelagem Molecular, Instituto de Ciências Exatas e Naturais, Universidade Federal do Pará CP 11101 60075-110 Belém PA Brazil fabioam@ufpa.br

## Abstract

The main protease (M^pro^ or 3CL^pro^) is a conserved cysteine protease from the coronaviruses and started to be considered an important drug target for developing antivirals, as it produced a deadly outbreak of COVID-19. Herein, we used a combination of drug reposition and computational modeling approaches including molecular docking, molecular dynamics (MD) simulations, and the calculated binding free energy to evaluate a set of drugs in complex with the M^pro^ enzyme. Particularly, our results show that darunavir and triptorelin drugs have favorable binding free energy (−63.70 and −77.28 kcal mol^−1^, respectively) in complex with the M^pro^ enzyme. Based on the results, the structural and energetic features that explain why some drugs can be repositioned to inhibit M^pro^ from SARS-CoV-2 were exposed. These features should be considered for the design of novel M^pro^ inhibitors.

## Abstract 2

Structural and energetic features explain why some drugs can be repositioned to inhibit M^pro^ from SARS-CoV-2.

## Graphical abstract

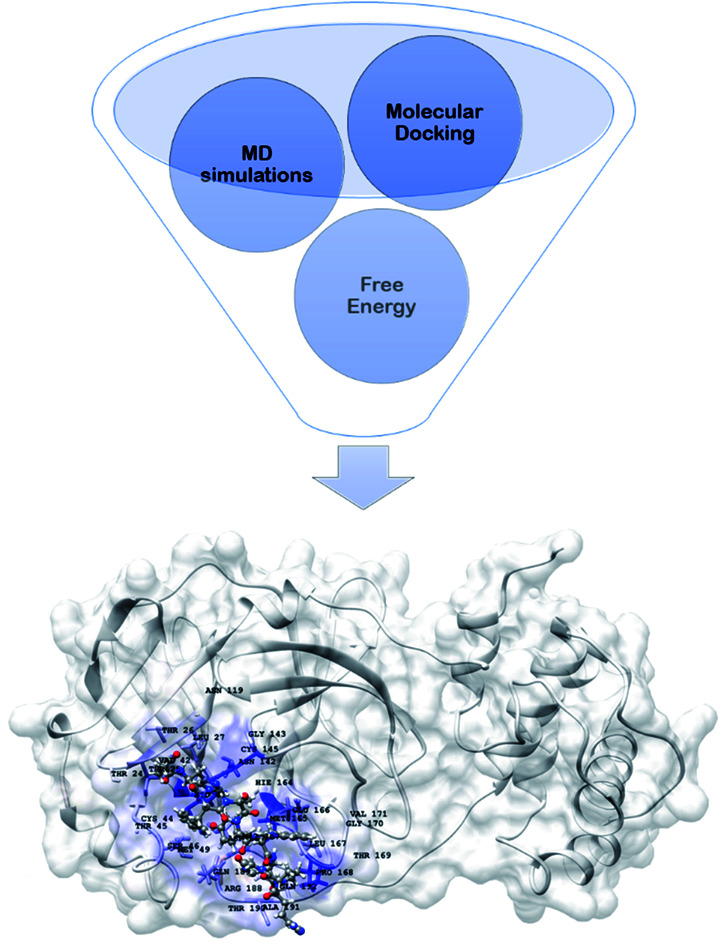

## Introduction

Coronaviruses (CoVs) are enveloped viruses with a diameter of 60 to 130 nm that contain a single-stranded positive ribonucleic acid (RNA) genome and belong to the Coronaviridae family, which is divided into four genera (α, β, γ e δ), in which SARS-CoV belongs to the genus Betacoronavirus.^1,2^

COVID-19 (COronaVIrus Disease 2019) is a disease caused by the SARS-CoV-2 coronavirus that has spread rapidly around the world since it was reported in late 2019 in Wuhan, China.^3^ SARS-CoV-2 is the seventh coronavirus known to infect humans and that can cause serious diseases, such as SARS-CoV and the Middle East respiratory syndrome (MERS-CoV),^4^ while HKU1,^5^ NL63,^6^ OC43 (ref. 7) and 229E^8^ are associated with mild symptoms. SARS-CoV-2 is the one with the highest transmission rate, as it includes asymptomatic carriers, a long latency period, and a high infection rate.^9^ COVID-19 causes fever, fatigue, dry cough, muscle pain, shortness of breath, and in some cases lead to pneumonia.^10^ In addition, a small proportion of those infected (∼5%) develop diseases that can progress to severe acute respiratory syndrome (SARS), which can lead to death.^9^ Since the initial SARS-CoV-2 outbreak, the World Health Organization (WHO) declared on March 11, 2020, COVID-19 as a pandemic disease due to the spread of the coronavirus.^11,12^ Until April 26, 2021, the total number of confirmed cases of COVID-19 was 146 841 882 with over 3 104 743 deaths in at least 223 countries,^13^ which indicates the epidemic is a serious public health problem.

The 3-chymotrypsin-like protease enzyme (3CL^pro^), also known as the “main protease” (M^pro^), is considered an attractive target in drug design for the treatment of coronavirus infection.^14,15^ The role of this protease involves the proteolytic processing of viral polyproteins, being considered essential in the process of replication and maturation of the virus.^16^ The M^pro^ cysteine protease contains three domains, domain I being formed by residues 1 to 101, and domain II consisting of antiparallel barrels that are formed by residues 102 to 184, and for enzyme activity domain III, composed mainly of alpha helices, formed by residues 201 to 301.^17,18^ The substrate-binding region is located in the gap between domains I and II and consists of a catalytic dyad formed by the residues Hys41 and Cys145, in which cysteine acts as a nucleophile and histidine as a proton acceptor.^14,19^ In addition, as M^pro^ does not have a homologous human enzyme, it presents itself as an ideal target for drug design.^15,19,20^

A traditional drug discovery process is expensive and time-consuming and can take decades to complete.^21^ Currently, there are no drugs available for the treatment of COVID-19, and in the absence of effective treatment, the redirection of drugs becomes an attractive solution, due to the reduction in development time and cost, as the drugs have already been tested to what concerns safety in humans, which means they should quickly enter the most advanced clinical stages.^22,23^

Structure-Based Virtual Screening (SBVS) is a method used to identify and prioritize ligands for subsequent *in vitro* and *in vivo* profiling and, therefore, is an attractive way to identify new compounds for more effective treatment of COVID-19.^18,24^ Therefore, in this study, a virtual screen based on the structure of the receptor in a set of molecules of approved drugs was used to identify promising candidates that could be tested against the SARS-CoV-2 enzyme. Then, molecular dynamics and binding free energy calculations were carried out to evaluate the inhibition potential of the selected commercial drugs against the M^pro^ enzyme of SARS-CoV-2. Overall, this work shines a light on the M^pro^–inhibitor complex interactions, which is paramount for the development of new drugs against COVID-19.

## Materials and methods

### Molecular docking, libraries description, and preparation

The crystallographic structure of the hydrolase enzyme was recovered from the Protein Data Bank (PDB) database under code 6LU7 with a resolution of 2.16 Å.^15^ The enzyme has a chain with a total of 306 amino acid residues. Molecular docking can be used to explore possible conformations of the ligand inside the receptor's binding pocket as well as estimation of the strength of ligand–protein interaction.^25^ The molecular docking simulations were performed using the GOLD 2020.1 program (Cambridge Crystallographic Data Centre – CCDC, Cambridge, UK).^26,27^ GOLD program uses a genetic algorithm to generate and select conformations of flexible compounds that bind to a protein's receptor site.^28^ Compounds were docked by applying the GoldScore scoring function with a search efficiency of 100%. The binding site was defined as a 10 Å sphere centered on the N3 crystallographic ligand. Before the preparation of the enzyme for docking, where the covalent bond between the Cys145 residue and the N3 crystallized ligand was removed. Finally, water molecules were removed from the structure of the enzyme, and hydrogen atoms were added by the GOLD program.

Before starting the virtual screen with all compounds chosen from the database, a re-docking of the N3 crystallographic ligand was carried out. Thus, the best conformation obtained by the program is compared with that of the ligand co-crystallized in the enzyme. In this way, an analysis is made by comparing the values of the root-mean-square deviation (RMSD). RMSD values below 2 Å show that the re-docking was successful according to data in the literature.^29^ The PoseView online server^30^ was used to analyze the interactions of hydrogens as well as hydrophobic interactions between the selected ligands and the amino acids of the enzyme, derived from molecular docking.

The two-dimensional structures of the 2998 compounds that were retrieved from the Repurposing hub (http://www.broadinstitute.org/repurposing) and built-in the MarvinSketch program,^31^ as well as all structures, were optimized in the OpenBabel program.^32^ For the selection of compounds, the total drug-score parameter calculated by the OSIRIS Property Explorer program was also evaluated.^33^ This measurement is a score generated by the combination of the parameters of drug-similarity, hydrophobicity (*c* log *P*), aqueous solubility (log *S*), molar mass, and toxicity risk data.^34^

### System setup for MD simulations

The molecular docking structures were submitted to QM optimization at the HF/6-31G* level by using the Gaussian09 program.^35^ Then, at the same QM level, partial atomic charges for all docked compounds were obtained by applying the RESP method^36^ as implemented in the antechamber module of the AmberTools19 package.^37^ The general AMBER force field (GAFF)^38^ and AMBER ff14SB^39^ were used in describing the MM parameters for the ligands and enzyme, respectively. The PROPKA3.0 (ref. 40) was used to compute the protonation states of the amino acid residues at neutral pH. Particularly, the catalytic dyad (Cys145 and His41) was considered in neutral form, as reported for the SARS-CoV-2 M^pro^.^41^ Then, the absent protons were added by using *tleap* module of the AmberTools18 package.^37^ Each system was solvated in a truncated octahedron box of TIP3P^42^ water molecules with a buffer region of at least 10 Å from the solute atoms. Then, Na+ ions were added to neutralize the overall charge of the complexes. The SHAKE algorithm^43^ was applied to constraint the covalent bonds involving hydrogen atoms. The particle mesh Ewald approach^44^ was adopted to compute the long-range electrostatic interactions, a cut-off distance of 10 Å was used.

Each resulting system was minimized using 10 000 steps of the steepest descent method followed by 5000 steps of the conjugate-gradient method until the root-mean-square of the gradient was below 10^−4^ kcal (mol^−1^·Å^−1^). After, each minimized system was heated up from 0 to 300 K with solute restrained using a harmonic potential with a force constant of 100 kcal (mol^−1^·Å^−2^) during 500 ps. Along the equilibration stage, the positional restraint force was reduced from 50 to 2 kcal (mol^−1^·Å^−2^) during 1.2 ns of MD simulations. Then, 200 ps of density equilibration without any restraint under the NPT was performed. Finally, 100 ns of NPT simulation at 300 K was performed with a 2 fs time step. The GPU version of the *pmemd*^45^ module of the Amber18 package was used to run all MM MD simulations. The structural analysis of each system was performed by computing the root-mean-square deviations (RMSD) of the main chain atoms concerning average coordinates structures. Similarly, the root-mean-square fluctuations (RMSF) of Cα atoms of each amino acid residue were computed. Besides, MD runs for M^pro^-triptorelin system were also performed with a different starting structure (random seed). In addition to that, additional replicas starting from different sets of atomic coordinates and velocities were also chosen to generate completely independent trajectories. Details are presented in the ESI† files. The enabled us to calculate the error for these free energy results.

### Binding free energy and residual decomposition analysis

For the binding free energy calculations, an average ensemble of structures from MD simulations, as described above, was extracted by using the CPPTRAJ module^46^ of the AmberTools18 package. A total of 1000 snapshots were extracted from the last 10 ns trajectory of the production MD stage (at 10 ps intervals). Next, MM/GBSA^47,48^ approach was employed to determine the binding free energy) M^pro^–ligand complexes using the MMPBSA.py,^49^ according to the eqn (1):

1 Δ*G*_bind_ = Δ*E*_MM_ + Δ*G*_SOLV_ − *T*Δ*S*

where, Δ*E*_MM_, gas-phase MM energy is computed by eqn (2):

2 Δ*E*_MM_ = Δ*E*_int_ + Δ*E*_ele_ + Δ*E*_vdW_

where, Δ*E*_int_, Δ*E*_ele_ and Δ*E*_vdW_ are the changes in the internal (bond, angles, and dihedral energies), electrostatic, and van der Waals energies, respectively.

The Δ*G*_SOLV_ is the sum of the electrostatic solvation energy (Δ*G*_GB_) (polar contribution) and the nonpolar contribution (Δ*G*_SA_), according to eqn (3):

3 Δ*G*_SOLV_ = Δ*G*_GB_ + Δ*G*_SA_

Particularly, Δ*G*_SA_ is estimated by using the solvent-accessible surface area (SASA) method.^50^ As the entropic term (−*T*Δ*S*) has a heavy computational cost, it has been neglected for binding free energy calculations.^51^

Finally, since the MM/PBSA^48,50^ approach is computationally time-consuming,^52^ MM/GBSA has been used to compute the contributions from individual residues by free energy decomposition analysis.^51^ This method has been frequently applied in drug design studies.^53–58^

## Results and discussion

### Virtual screening and molecular docking calculation

It should be highlighted that SARS-CoV-2 M^pro^ is a crucial target for potential repurposing of known clinical drugs^41,59–64^ as well as for designing specific protease inhibitors.^15,65–67^ Although clinical drugs are not available for use against SARS-CoV-2 M^pro^, others protease inhibitors have been designed to inhibit the very closely related SARS-CoV M^pro^.^1,68–70^ Therefore, in this study, a computational protocol was applied to provide new insights for structure-assisted and computational drug design against SARS-CoV-2 M^pro^.

Structure-based virtual screening was carried out in the gold program 2020.1, which uses a genetic algorithm to generate and select conformers of the database of compounds. From the results of re-docking, it was found that re-docking of the crystallographic ligand with the gold program gives an RMSD value of 2 Å. Thus, it is observed that the GOLD program was able to reproduce the conformation of the crystallographic ligand and the main interactions obtained at the M^pro^ enzyme site.^29^ Besides, molecular docking calculations have been successfully applied in recent SARS-CoV-2 M^pro^ studies.^71–75^

Initially, 75 of the nearly 3000 drug molecules that had the best scores in the GOLD program were selected. It is noteworthy that the selection criterion was based on the score of remdesivir, as this has a potential effect in the treatment of COVID-19.^9,29,76^ After, a visual inspection of the docking results was carried out on the Poseview online server,^30^ taking into account mainly the interactions with the catalytic dyad residues of the M^pro^ enzyme, in addition to the drug-score values to qualify the best drug candidates against COVID-19.^77^ Thus, from the 75 structures, ten hit compounds were selected for further analysis (Table 1), considering the highest gold score values with M^pro^ enzyme. Besides, they were selected using as criteria hydrogen interactions, complementarity through visual analysis at the active site of the M^pro^ enzyme and the drug-score values.

**Table 1:** The selected compounds, GOLD score values, hydrogen, and hydrophobic interactions, drug score values, and pharmacological activity

Compound	Gold score	Hydrogen interactions	Hydrophobic interactions	Drug score	Pharmacological activity
Afamelanotide	111.02	Asn119, 2 × (Asn142), His164, Gln189	**His41**, Leu141, Asn142, Gly143, Met165, Gln189	0.31	Erythropoietic protoporphyria
Triptorelin	110.38	2 × (Asn142), Glu166, Pro168, Asp187, Gln189	Thr25, Ser46, Met49, Asn142, **Cys145**, His163, Met165, Glu166, Gln189	0.33	Antineoplastic, synthetic analog of gonadotropin-releasing hormone palliative treatment of advanced prostate cancer. Stimulates the release of luteinizing hormone
Pentagastrin	98.06	Gly143, Ser144, His163, Glu166, Gln189	Asn142, Met165, Pro168, Gln189, Thr190, Ala191	0.18	Evaluation of gastric acid secretory function
Terlipressin	97.51	Thr24, Ser139, Phe140, 2 × (Asn142), Gly143, Met165, 2 × (Glu166), Gly170	Thr25, Ser46, Met49, His172	0.46	Vasoactive drug in the management of hypotension
Adaptavir	92.78	Thr24, Thr26, Ser46, Thr190, Gly143, 2 × (**Cys145**), Thr190, Gln192	Met165	0.50	Chemokine receptor antagonist
Pepstatin	90.27	Thr24, 2 × (Thr26), **His41**, Cys145, Gln189	**Cys145**	0.44	Inhibitor of aspartic proteinases
Octreotide	88	Ser46, Met49, **Cys145**, Glu166	**His41**, Ser46, Met49, Glu166, Pro168, Gln189, Thr190,	0.21	Potent inhibitor of growth hormone, glucagon, and insulin
Darunavir	85.46	**Cys145**, 2 × (Ser144), 2 × (Glu166), Gln189	**His41**, Met49, Leu141, Asn142, Met165, Gln189	0.29	Protease inhibitor used as a treatment of human immunodeficiency virus (HIV)
Afatinib	84.43	His41, 2 × (Glu166), Gln189	**His41**, **Cys145**, Met165, Asp187	0.24	Therapy of selected forms of metastatic non-small cell lung cancer
Foretinib	80.31	Thr26, **Cys145**	Thr25, Thr26, **His41**, Met49, Leu50, Gly143, Met165, Gln189	0.25	Treatment of breast cancer

In addition to the dyad, the enzyme M^pro^ has a catalytic region formed by the residues Phe140, Asn142, Gly143, Ser144, Cys145, Met165, Glu166, Gln189 and Thr190.^78^ As a protease, the catalytic site contains different subsites where interactions occur with the amino acids of the substrate whose peptide bond will be hydrolyzed. The S1 subsite is formed by the residues Phe140, Leu141, Asn142, His163 and Glu166, the S2 subsite is formed by the residues Met49, Tyr54, His164, Asp187 and Arg188, the S3 subsite is formed by the residues Met165, Leu167, Gln189, Thr190 and Gln192, in addition to the S1′, formed by the residues His41, Gly143, Ser144 and Cys145.^75^

Analyzing Table 1, it was possible to observe that the selected ligands interact with the enzyme through hydrogen bonds with the Thr24, Thr26, Hys41, Ser46, Met49, Asn119, Ser139, Phe140, Asn142, Gly143, Ser144, Cys145, His163, His164, Met165, Glu166, Pro168, Gly170, Asp187, Gln189, Thr190 and Gln192. In addition, among the ten main compounds selected, the interactions of residues His41, Asn142, Cys145, Glu166 and Gln189 can be highlighted, in which they make two, three, five, six and six hydrogen bonds, respectively. As mentioned earlier, His41 and Cys145 are part of the catalytic dyad, and a computational study of Wang's drug repurposing showed that His41 and Gln189 residues were considered important.^79^ These residues are mainly part of the S1 subsite (Asn142, Cys145 e Glu166) and S2 (His41 e Gln189), in addition, studies by Koulgi and collaborators suggest that the inhibitory effect is observed when interactions with these key residues occur at the active site of the enzyme.^80^

Apart from remdesivir, application of darunavir against Covid-19 should be highlighted, especially since it has been studied as a possible treatment for SARS-CoV-2, and due to *in vitro* evidence that supports its ability to fight the disease.^81^

### Stability of MD simulations

**Fig. 1:**
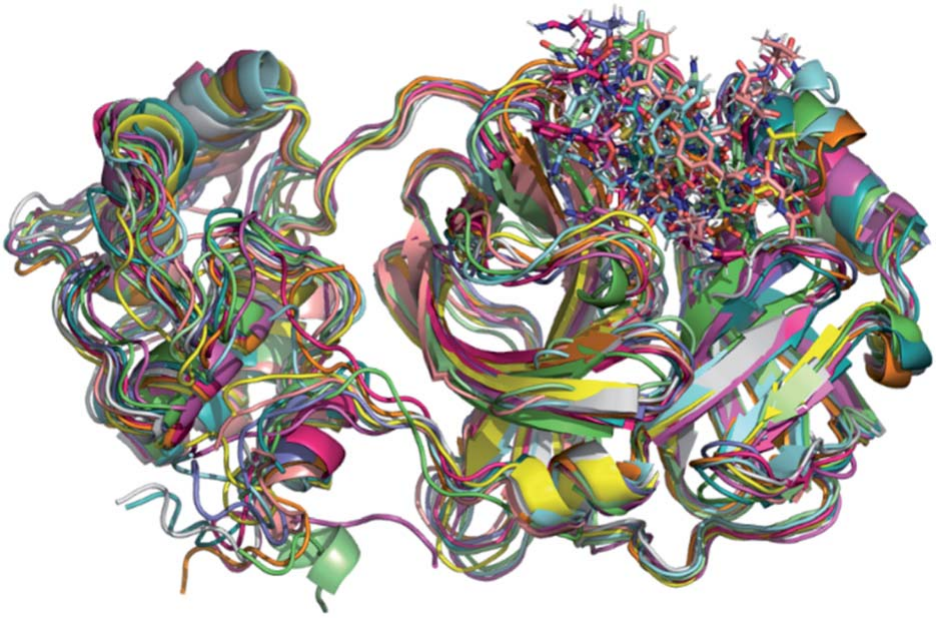
Overlay of all M^pro^ systems after 100 ns of MD simulations. M^pro^ is shown in the cartoon model while repurposed drugs are in the stick model. All 3D structures of representative snapshots of M^pro^ systems are available as ESI† (PDB format).

Initially, it should be noted that experimental evidence reveals that in the biological environment, M^pro^ enzyme acts in the dimer form instead of the monomeric one.^82^ However, for computational proposals, it is possible to speed up computer-aided drug design by using only the monomeric form of SARS-CoV-2 M^pro^.^73^ Therefore, a total of 100 ns production simulations were performed for each repurposed drug on the M^pro^ enzyme from molecular docking procedures. Initially, we compare the representative snapshots chosen from the last 10 ns MD trajectory of each system. As illustrated in Fig. 1, the binding modes of all repurposed drugs in the active sites of the M^pro^ from MD simulated results are nearly the same place.

**Fig. 2:**
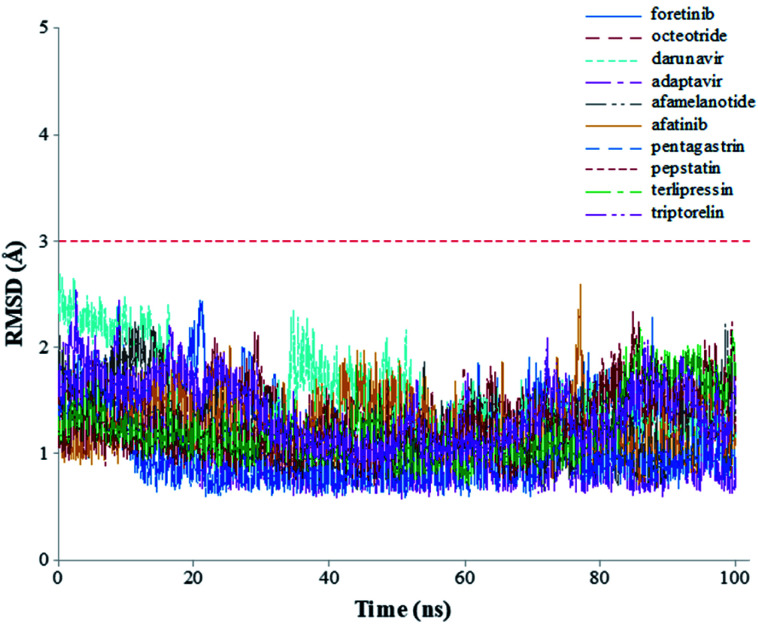
Graphical representation of the RMSD values (Å) during 100 ns of MD simulation time for the selected repurposed drug complexes.

The root-mean-square deviations (RMSDs) of each system concerning the average structure were plotted in Fig. 2. As the plots show, the RMSDs of each system tend to converge after 20 ns simulation time, indicating the system is stable and equilibrated. Besides, all systems show very small RMSDs, which change from 0.97 ± 0.23 Å (pentagastrin) to 1.40 ± 0.20 Å (octeotride), which suggests that they are in a similar conformation. Additionally, for the M^pro^-triptorelin, which shows the best binding free energy (as shown below), two more 100 ns of MD runs were performed using random seed with different atomic velocities. These results suggest that their average structures have very high 3D conformations and similar RMSD plots (Fig. S1 in the ESI† file).

**Fig. 3:**
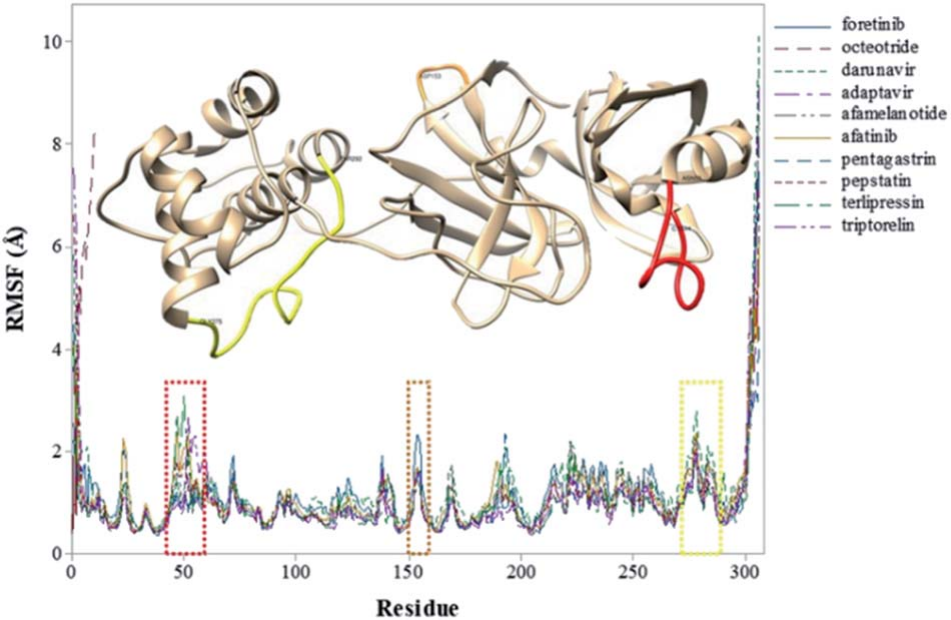
RMSF values (in Å) of M^pro^–inhibitor complexes.

To explore the fluctuations of the residue of system-wise, the root-mean-square fluctuation (RMSF) analysis was carried out. The RMSF of the Cα atoms of the protein for each residue during whole MD simulations for the complexes is shown in Fig. 3. The highest RMSF values are attributed to the N- and C-terminals, and loop regions. As shown in Fig. 3, the region closer to the active site formed by the Cys44–Asn53 residues (represented in red), that presented greater fluctuation to the terlipressin compound. Based on this observation, Bzówka *et al.* suggested that the presence of an inhibitor in this region might stabilize the loops surrounding the active site.^72^ Another highlight is that residues are formed by the Asp153–Cys156 (represented in orange). In addition, a study by Shekh and colleagues describes the importance of Cys156 in the accessibility of external agents in the M^pro^ enzyme.^83^ Another region that presented high values of RMSF (Fig. 3), for all compounds, was the region formed by the Gly275–Thr292 residues (represented in yellow).

### Binding free energy analysis

The binding free energies were computed using MM/GBSA method^48^ as implemented in MMPBSA.py^49^ are presented in Table 2. As a single MD trajectory of the bound complexes was used to compute Δ*G*_bind_, the Δ*E*_int_ term can be canceled once the energy difference between complex systems and their components are computed using the same configurations.^51^

**Table 2:** MM/GBSA binding free energies (Δ*G*_bind_) and its components for the repurposed drugs in the M^pro^ enzyme. The errors were computed by using single-trajectory protocol.^48^ All values are reported in kcal mol^−1^

Inhibitor	Δ*E*_ele_	Δ*E*_vdW_	Δ*G*_GB_	Δ*G*_SA_	Δ*G*_bind_
Triptorelin	−121.9 ± 0.3	−92.4 ± 0.2	147.9 ± 0.3	−10.8 ± 0.01	−77.3 ± 0.2
Darunavir	−207.7 ± 0.7	−67.7 ± 0.2	221.9 ± 0.6	−10.2 ± 0.01	−63.7 ± 0.3
Foretinib	−104.1 ± 0.4	−63.6 ± 0.2	122.2 ± 0.4	−7.0 ± 0.01	−52.5 ± 0.2
Pentagastrin	24.8 ± 0.5	−57.9 ± 0.2	−7.9 ± 0.4	−7.3 ± 0.02	−48.2 ± 0.2
Adaptavir	−34.5 ± 0.3	−53.3 ± 0.2	51.6 ± 0.3	−6.1 ± 0.02	−42.2 ± 0.2
Afamelanotide	−185.0 ± 1.4	−57.8 ± 0.2	211.2 ± 1.3	−7.7 ± 0.03	−39.4 ± 0.3
Afatinib	−115.7 ± 0.5	−51.3 ± 0.1	134.6 ± 0.5	−6.6 ± 0.01	−38.9 ± 0.1
Octeotride	−243.1 ± 1.0	−43.1 ± 0.2	261.7 ± 0.9	−6.1 ± 0.02	−30.5 ± 0.2
Pepstatin	42.9 ± 0.4	−49.8 ± 0.2	−15.9 ± 0.3	−6.4 ± 0.02	−29.2 ± 0.2
Terlipressin	−220.2 ± 1.4	−54.2 ± 0.3	256.5 ± 1.6	−7.4 ± 0.04	−25.4 ± 0.2

The energy components of the binding free energies were also listed in Table 2. As can be seen from Table 2, the Δ*E*_ele_, Δ*E*_vdW_ and Δ*G*_SA_terms are favorable contributors to most of the repurposed drugs, except for the pentagastrin and pepstatin drugs. Interestingly, in these systems, the term Δ*G*_GB_ is favorable, which does not occur on other systems. Then, in most of the systems, the favorable electrostatic interactions are equilibrated by the unfavorable polar solvation upon binding. As a result, the total electrostatic interaction contributions have detrimental effects on the binding of repurposed drugs. Except for the pentagastrin and pepstatin, the positive solvation contribution indicates that the repurposed drugs were exposed to water interaction in their respective complexes. If we consider the contributions of different binding free energy components of M^pro^–ligand binding, the most important determinant of difference in the binding affinity are Δ*E*_vdW_ and Δ*G*_GB_ contributions.

Interestingly, according to molecular docking results, the triptorelin was pointed with the second-highest value of gold score (Table 1) and after 100 ns of MD simulations has become the most promising M^pro^ inhibitor among the selected compounds. On the other hand, terlipressin, which has the fourth-highest value of gold score (Table 1), has been identified as the weakest M^pro^ inhibitor. This evidence can be observed by comparing the binding free energy values of the selected repurposed drugs (Table 2) it is possible to observe two compounds with distinct energy values. The calculated values of the terlipressin and triptorelin compounds have Δ*G*_bind_ (−25.39 ± 0.21 and −77.28 ± 0.20 kcal mol^−1^, respectively) and Δ*E*_vdW_ contribution (−54.24 ± 0.30 and −92.42 ± 0.19 kcal mol^−1^, respectively). These results suggest the importance of applied computational strategies as highlighted in previous studies.^25^

**Fig. 4:**
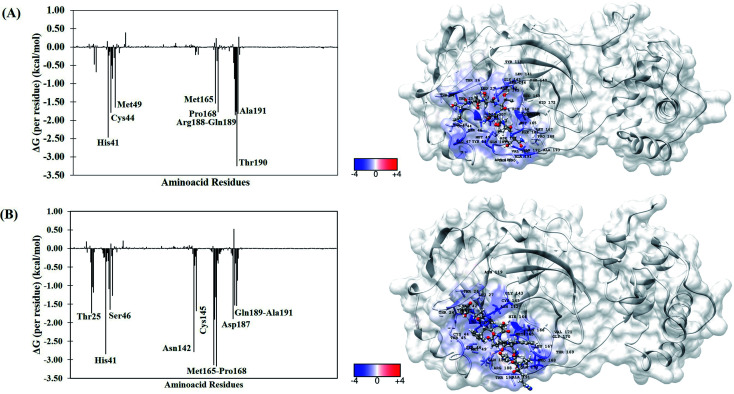
Per-residue binding free energy decomposition (in kcal mol^−1^) (left) and representative snapshot (right) of M^pro^ systems after 100 ns of MD simulations. (A) Terlipressin and (B) triptorelin. The carbon atoms of each inhibitor are shown in gray color. The per-residue results for other systems are available in the ESI file (Fig. S2†).

To better understand the individual contribution of each residue for the binding free energy for the lowest Δ*G*_bind_, a residual decomposition analysis of Δ*G*_bind_ was performed using MM/GBSA approach.^51^ Here, any residue that contributes to the binding free energy values below −1.50 kcal mol^−1^ was considered an important residue to the binding process. The energy contribution of each residue for the complexes formed by terlipressin (weakest binding free energy) and triptorelin (strongest binding free energy) obtained by the MM/GBSA method are shown in Fig. 4, once they have shown the different binding free energy values. The residue contributions to binding energies using the plugin CHEWD^84^ with Chimera^85^ software are shown on the right side of Fig. 4. From this, the favourable interactions are at the blue of the colour scale, that is, those that contribute to the stabilization of ligand into complex, while the red colour represents interaction by residue with positive values and corresponds to unfavourable interaction values.

For the M^pro^–terlipressin complex (Fig. 4A) the residues that most significantly contribute to the total energy and, therefore, to the stabilization of the complex are His41, Cys44, Met49, Met165, Pro168, Pro188, Gln189, Thr190 and Ala191, that displayed energy values of −2.46, −1.79, −1.65, −1.53, −1.77, −1.86, −1.75, −3.25 and −1.82 kcal mol^−1^, respectively. The binding free energy decomposition of M^pro^–triptorelin complex (Fig. 4B) revealed that the residues Thr25, His41, Ser46, Asn142, Cys145, Met165, Glu166, Pro168, Asp187, Gln189 and Ala191 contribute greatly to the complex stability, exhibiting the energy values −1.74, −2.85, −1.65, −2.78, −1.69, −3.14, −1.91, −3.18, −1.90, −1.53 and −1.54 kcal mol^−1^, respectively. From these results, we can observe that the enzyme–triptorelin complex present more favorable contributions to the binding free energy when compared to those formed by the terlipressin complex. Moreover, we should pay more attention to those residues with a relatively large difference in the contribution to the total binding free energies. For the terlipressin system, we can highlight the residues His41 and Thr190, while for the triptorelin system, the most relevant residues are His41, Asn142, Met165, and Pro168.

It is worth highlighting that His41, Met165, Pro168, Gln189 and Ala191 residues were important for both ligands. Interestingly, that triptorelin compound has interactions with catalytic dyad formed by His41 and Cys145 residues.^15^ Besides, the interactions with residuals Thr25, Cys145, Met165, Asp187, Gln189 and Gln192 had already been observed in the molecular docking results (Table 1).

Particularly, we can highlight the results for the darunavir, which shows a moderate binding free energy value (−63.70 ± 0.25 kcal mol^−1^). This drug has shown evidence *in vitro* studies involving a possible treatment for SARS-CoV-2.^86^ Therefore, our results suggest that M^pro^ would be a potential target for the darunavir drug.

## Conclusion

Drug repurposing is the well-established safety strategy of the development of novel therapeutic compounds, where the principal advantage is reducing the time and cost of new candidates in a future clinical trial. The COVID-19 caused by the SARS-CoV-2 has become a pandemic health crisis and the discovery of effective treatment against the virus remains an outstanding challenge. The results of this study showed that the use of molecular modelling approaches, such as molecular docking and molecular dynamics simulations, can be used to design new drug leads that specifically target the M^pro^ enzyme from SARS-CoV-2. The molecular docking results allowed the identification of key interactions in the M^pro^ active site that make hydrogens bonds involving His41, Asn142, Cys145, Glu166 and Gln189 residues. The MD simulations of the best top 10 compounds indicated that systems were stable and equilibrated along the trajectory in the active site of the M^pro^ enzyme, as well as a favourable value for the prediction of the binding energy with the MM-GBSA method. It is worth mentioning that the darunavir and triptorelin drugs exhibited the strongest binding free energies in complex with M^pro^ enzyme. Thus, darunavir and triptorelin are potential inhibitors of SARS-CoV-2. In summary, all of the repurposed compounds reported here may provide insights for structure-assisted optimization to for potential application against the COVID-19 pandemic.

## Author contributions

The manuscript was written through the contributions of all authors. All authors have approved the final version of the manuscript.

## Conflicts of interest

There are no conflicts to declare.

## Supplementary Material

RA-011-D1RA03956C-s001

RA-011-D1RA03956C-s002

RA-011-D1RA03956C-s003

RA-011-D1RA03956C-s004

RA-011-D1RA03956C-s005

RA-011-D1RA03956C-s006

RA-011-D1RA03956C-s007

RA-011-D1RA03956C-s008

RA-011-D1RA03956C-s009

RA-011-D1RA03956C-s010

RA-011-D1RA03956C-s011

## References

[cit1] Hamre D., Procknow J. J. (1966). Exp. Biol. Med..

[cit2] Schoeman D., Fielding B. C. (2019). Virol. J..

[cit3] Zhu N., Zhang D., Wang W., Li X., Yang B., Song J., Zhao X., Huang B., Shi W., Lu R., Niu P., Zhan F., Ma X., Wang D., Xu W., Wu G., Gao G. F., Tan W. (2020). N. Engl. J. Med..

[cit4] Drosten C., Günther S., Preiser W., van der Werf S., Brodt H.-R., Becker S., Rabenau H., Panning M., Kolesnikova L., Fouchier R. A. M., Berger A., Burguière A.-M., Cinatl J., Eickmann M., Escriou N., Grywna K., Kramme S., Manuguerra J.-C., Müller S., Rickerts V., Stürmer M., Vieth S., Klenk H.-D., Osterhaus A. D. M. E., Schmitz H., Doerr H. W. (2003). N. Engl. J. Med..

[cit5] Woo P. C. Y., Lau S. K. P., Chu C., Chan K., Tsoi H., Huang Y., Wong B. H. L., Poon R. W. S., Cai J. J., Luk W., Poon L. L. M., Wong S. S. Y., Guan Y., Peiris J. S. M., Yuen K. (2005). J. Virol..

[cit6] van der Hoek L., Pyrc K., Jebbink M. F., Vermeulen-Oost W., Berkhout R. J. M., Wolthers K. C., Wertheim-van Dillen P. M. E., Kaandorp J., Spaargaren J., Berkhout B. (2004). Nat. Med..

[cit7] Hamre D., Procknow J. J. (1966). Exp. Biol. Med..

[cit8] McIntosh K., Dees J. H., Becker W. B., Kapikian A. Z., Chanock R. M. (1967). Proc. Natl. Acad. Sci. U. S. A..

[cit9] Guy R. K., DiPaola R. S., Romanelli F., Dutch R. E. (2020). Science.

[cit10] Guan W., Ni Z., Hu Y., Liang W., Ou C., He J., Liu L., Shan H., Lei C., Hui D. S. C., Du B., Li L., Zeng G., Yuen K.-Y., Chen R., Tang C., Wang T., Chen P., Xiang J., Li S., Wang J., Liang Z., Peng Y., Wei L., Liu Y., Hu Y., Peng P., Wang J., Liu J., Chen Z., Li G., Zheng Z., Qiu S., Luo J., Ye C., Zhu S., Zhong N. (2020). N. Engl. J. Med..

[cit11] Arshad Ali S., Baloch M., Ahmed N., Arshad Ali A., Iqbal A. (2020). Journal of Infection and Public Health.

[cit12] Chakraborty I., Maity P. (2020). Sci. Total Environ..

[cit13] WHO , Coronavirus Disease (COVID-19) Outbreak Situation, https://covid19.who.int/

[cit14] Yang H., Yang M., Ding Y., Liu Y., Lou Z., Zhou Z., Sun L., Mo L., Ye S., Pang H., Gao G. F., Anand K., Bartlam M., Hilgenfeld R., Rao Z. (2003). Proc. Natl. Acad. Sci. U. S. A..

[cit15] Jin Z., Du X., Xu Y., Deng Y., Liu M., Zhao Y., Zhang B., Li X., Zhang L., Peng C., Duan Y., Yu J., Wang L., Yang K., Liu F., Jiang R., Yang X., You T., Liu X., Yang X., Bai F., Liu H., Liu X., Guddat L. W., Xu W., Xiao G., Qin C., Shi Z., Jiang H., Rao Z., Yang H. (2020). Nature.

[cit16] Nutho B., Mahalapbutr P., Hengphasatporn K., Pattaranggoon N. C., Simanon N., Shigeta Y., Hannongbua S., Rungrotmongkol T. (2020). Biochemistry.

[cit17] Gentile D., Patamia V., Scala A., Sciortino M. T., Piperno A., Rescifina A. (2020). Mar. Drugs.

[cit18] Mittal L., Kumari A., Srivastava M., Singh M., Asthana S. (2020). J. Biomol. Struct. Dyn..

[cit19] Dai W., Zhang B., Jiang X.-M., Su H., Li J., Zhao Y., Xie X., Jin Z., Peng J., Liu F., Li C., Li Y., Bai F., Wang H., Cheng X., Cen X., Hu S., Yang X., Wang J., Liu X., Xiao G., Jiang H., Rao Z., Zhang L.-K., Xu Y., Yang H., Liu H. (2020). Science.

[cit20] Bzówka M., Mitusińska K., Raczyńska A., Samol A., Tuszyński J. A., Góra A. (2020). Int. J. Mol. Sci..

[cit21] Rang H. P. and Hill R. G., in Drug Discovery and Development, ed. R. G. Hill and H. P. Rang, Churchill Livingstone, 2nd edn, 2013, pp. 203–209

[cit22] Liu Z., Fang H., Reagan K., Xu X., Mendrick D. L., Slikker W., Tong W. (2013). Drug Discovery Today.

[cit23] Schein C. H. (2020). Med. Res. Rev..

[cit24] Araujo S. C., Maltarollo V. G., Almeida M. O., Ferreira L. L. G., Andricopulo A. D., Honorio K. M. (2020). Molecules.

[cit25] Honarparvar B., Govender T., Maguire G. E. M., Soliman M. E. S., Kruger H. G. (2014). Chem. Rev..

[cit26] Jones G., Willett P., Glen R. C., Leach A. R., Taylor R. (1997). J. Mol. Biol..

[cit27] Verdonk M. L., Cole J. C., Hartshorn M. J., Murray C. W., Taylor R. D. (2003). Proteins: Struct., Funct., Bioinf..

[cit28] Jones G., Willett P., Glen R. C. (1995). J. Mol. Biol..

[cit29] Hevener K. E., Zhao W., Ball D. M., Babaoglu K., Qi J., White S. W., Lee R. E. (2009). J. Chem. Inf. Model..

[cit30] Stierand K., Rarey M. (2010). J. Cheminf..

[cit31] ChemAxon, 2021

[cit32] O'Boyle N. M., Banck M., James C. A., Morley C., Vandermeersch T., Hutchison G. R. (2011). J. Cheminf..

[cit33] Sander T., Freyss J., von Korff M., Reich J. R., Rufener C. (2009). J. Chem. Inf. Model..

[cit34] Azam F., Amer A., Abulifa A., Elzwawi M. (2014). Drug Des., Dev. Ther..

[cit35] Frisch G. M. J. , Trucks W., Schlegel H. B., Scuseria G. E., Robb M. A., Cheeseman J. R., Scalmani G., Barone V., Mennucci B., Petersson G. A., Nakatsuji H., Caricato M., Li X., Hratchian H. P., Izmaylov A. F., Bloino J., Zheng G., Sonnenberg J. L., Frisch M. J., Trucks G. W., Schlegel H. B., Scuseria G. E., Robb M. A., Cheeseman G., Scalmani J. R., Barone V., Mennucci B., Petersson H., Nakatsuji G. A., Caricato M., Li X., Hratchian H. P., Izmaylov A. F., Bloino J., Zheng G., Sonnenberg J. L., Hada K., Ehara M., Toyota M., Fukuda R., Hasegawa J., Ishida M., Nakajima T., Honda Y., Kitao O., Nakai H., Vreven T., Montgomery J. E., Peralta Jr J. A., Ogliaro F., Bearpark M., Heyd J. J., Brothers E., Kudin K. N., Staroverov R., Kobayashi V. N., Normand K., Raghavachari J., Rendell A., Burant J. C., Iyengar S. S., Tomasi J., Cossi M., Rega N., Millam J. M., Klene M., Knox J. E., Cross J. B., Bakken V., Adamo C., Jaramillo J., Gomperts R., Stratmann R. E., Yazyev O., Austin A. J., Cammi R., Pomelli C., Ochterski J. W., Martin R. L., Morokuma K., Zakrzewski V. G., Voth G. A., Salvador P., Dannenberg S., Dapprich J. J., Daniels A. D., Farkas Ö., Foresman J. B., Ortiz J. V., Cioslowski J. and Fox D. J., Gaussian 09, Revision E. 01, Gaussian, 2009

[cit36] Bayly C. I., Cieplak P., Cornell W., Kollman P. A. (1993). J. Phys. Chem..

[cit37] Case D. A. , Ben-Shalom I. Y., Brozell S. R., Cerutti D. S., Cheatham III T. E., Cruzeiro V. W. D., Darden T. A., Duke R. E., Ghoreishi D., Gilson M. K., Gohlke H., Goetz A. W., Greene D., Harris R., Homeyer N., Huang Y., Izadi S., Kovalenko A., Kurtzman T., Lee T. S., LeGrand S., Li P., Lin C., Liu J., Luchko T., Luo R., Mermelstein D. J., Merz K. M., Miao Y., Monard G., Nguyen C., Nguyen H., Omelyan I., Onufriev A., Pan F., Qi R., Roe D. R., Roitberg A., Sagui C., Schott-Verdugo S., Shen J., Simmerling C. L., Smith J., Salomon-Ferrer R., Swails J., Walker R. C., Wang J., Wei H., Wolf R. M., Wu X., Xiao L., York D. M. and Kollman P. A., Amber 2018, University of California, San Francisco, 2018

[cit38] Wang J., Wolf R. M., Caldwell J. W., Kollman P. A., Case D. A. (2004). J. Comput. Chem..

[cit39] Maier J. A., Martinez C., Kasavajhala K., Wickstrom L., Hauser K. E., Simmerling C. (2015). J. Chem. Theory Comput..

[cit40] Olsson M. H. M., Søndergaard C. R., Rostkowski M., Jensen J. H. (2011). J. Chem. Theory Comput..

[cit41] Kneller D. W., Phillips G., Weiss K. L., Zhang Q., Coates L., Kovalevsky A. (2021). J. Med. Chem..

[cit42] Jorgensen W. L., Chandrasekhar J., Madura J. D., Impey R. W., Klein M. L. (1983). J. Chem. Phys..

[cit43] Ryckaert J.-P., Ciccotti G., Berendsen H. J. C. (1977). J. Comput. Phys..

[cit44] Darden T., York D., Pedersen L. (1993). J. Chem. Phys..

[cit45] Salomon-Ferrer R., Götz A. W., Poole D., Le Grand S., Walker R. C. (2013). J. Chem. Theory Comput..

[cit46] Roe D. R., Cheatham T. E. (2013). J. Chem. Theory Comput..

[cit47] Srinivasan J., Cheatham T. E., Cieplak P., Kollman P. A., Case D. A. (1998). J. Am. Chem. Soc..

[cit48] Wang E., Sun H., Wang J., Wang Z., Liu H., Zhang J. Z. H., Hou T. (2019). Chem. Rev..

[cit49] Miller B. R., McGee T. D., Swails J. M., Homeyer N., Gohlke H., Roitberg A. E. (2012). J. Chem. Theory Comput..

[cit50] Kollman P. A., Massova I., Reyes C., Kuhn B., Huo S., Chong L., Lee M., Lee T., Duan Y., Wang W., Donini O., Cieplak P., Srinivasan J., Case D. A., Cheatham T. E. (2000). Acc. Chem. Res..

[cit51] Genheden S., Ryde U. (2015). Expert Opin. Drug Discovery.

[cit52] Genheden S., Ryde U. (2011). J. Chem. Theory Comput..

[cit53] Ahmed S. M., Maguire G. E. M., Kruger H. G., Govender T. (2014). Chem. Biol. Drug Des..

[cit54] Ntombela T., Fakhar Z., Ibeji C. U., Govender T., Maguire G. E. M., Lamichhane G., Kruger H. G., Honarparvar B. (2018). J. Comput.-Aided Mol. Des..

[cit55] Sanusi Z. K., Lawal M. M., Govender T., Maguire G. E. M., Honarparvar B., Kruger H. G. (2019). J. Phys. Chem. B.

[cit56] Souza A., Cardoso F., Martins L., Alves C., Silva J., Molfetta F. (2021). J. Braz. Chem. Soc..

[cit57] Pereira P. R. M., Araújo J. d. O., Silva J. R. A., Alves C. N., Lameira J., Lima A. H. (2020). J. Chem. Inf. Model..

[cit58] Martins L. S., Lameira J., Kruger H. G., Alves C. N., Silva J. R. A. (2020). Int. J. Mol. Sci..

[cit59] Ferraz W. R., Gomes R. A., S Novaes A. L., Goulart Trossini G. H. (2020). Future Med. Chem..

[cit60] Cavasotto C. N., Di Filippo J. I. (2021). Mol. Inf..

[cit61] Touret F., Gilles M., Barral K., Nougairède A., van Helden J., Decroly E., de Lamballerie X., Coutard B. (2020). Sci. Rep..

[cit62] Meyer-Almes F.-J. (2020). Comput. Biol. Chem..

[cit63] Riva L., Yuan S., Yin X., Martin-Sancho L., Matsunaga N., Pache L., Burgstaller-Muehlbacher S., De Jesus P. D., Teriete P., V Hull M., Chang M. W., Chan J. F.-W., Cao J., Poon V. K.-M., Herbert K. M., Cheng K., Nguyen T.-T. H., Rubanov A., Pu Y., Nguyen C., Choi A., Rathnasinghe R., Schotsaert M., Miorin L., Dejosez M., Zwaka T. P., Sit K.-Y., Martinez-Sobrido L., Liu W.-C., White K. M., Chapman M. E., Lendy E. K., Glynne R. J., Albrecht R., Ruppin E., Mesecar A. D., Johnson J. R., Benner C., Sun R., Schultz P. G., Su A. I., García-Sastre A., Chatterjee A. K., Yuen K.-Y., Chanda S. K. (2020). Nature.

[cit64] Ma C., Sacco M. D., Hurst B., Townsend J. A., Hu Y., Szeto T., Zhang X., Tarbet B., Marty M. T., Chen Y., Wang J. (2020). Cell Stress.

[cit65] Douangamath A., Fearon D., Gehrtz P., Krojer T., Lukacik P., Owen C. D., Resnick E., Strain-Damerell C., Aimon A., Ábrányi-Balogh P., Brandão-Neto J., Carbery A., Davison G., Dias A., Downes T. D., Dunnett L., Fairhead M., Firth J. D., Jones S. P., Keeley A., Keserü G. M., Klein H. F., Martin M. P., Noble M. E. M., O'Brien P., Powell A., Reddi R. N., Skyner R., Snee M., Waring M. J., Wild C., London N., von Delft F., Walsh M. A. (2020). Nat. Commun..

[cit66] Zhang L., Lin D., Sun X., Curth U., Drosten C., Sauerhering L., Becker S., Rox K., Hilgenfeld R. (2020). Science.

[cit67] Jin Z., Zhao Y., Sun Y., Zhang B., Wang H., Wu Y., Zhu Y., Zhu C., Hu T., Du X., Duan Y., Yu J., Yang X., Yang X., Yang K., Liu X., Guddat L. W., Xiao G., Zhang L., Yang H., Rao Z. (2020). Nat. Struct. Mol. Biol..

[cit68] Konno S., Thanigaimalai P., Yamamoto T., Nakada K., Kakiuchi R., Takayama K., Yamazaki Y., Yakushiji F., Akaji K., Kiso Y., Kawasaki Y., Chen S.-E., Freire E., Hayashi Y. (2013). Bioorg. Med. Chem..

[cit69] Jacobs J., Grum-Tokars V., Zhou Y., Turlington M., Saldanha S. A., Chase P., Eggler A., Dawson E. S., Baez-Santos Y. M., Tomar S., Mielech A. M., Baker S. C., Lindsley C. W., Hodder P., Mesecar A., Stauffer S. R. (2013). J. Med. Chem..

[cit70] Liu Y., Liang C., Xin L., Ren X., Tian L., Ju X., Li H., Wang Y., Zhao Q., Liu H., Cao W., Xie X., Zhang D., Wang Y., Jian Y. (2020). Eur. J. Med. Chem..

[cit71] Shah B., Modi P., Sagar S. R. (2020). Life Sci..

[cit72] Bzówka M., Mitusińska K., Raczyńska A., Samol A., Tuszyński J. A., Góra A. (2020). Int. J. Mol. Sci..

[cit73] Tam N. M., Nam P. C., Quang D. T., Tung N. T., V Vu V., Ngo S. T. (2021). RSC Adv..

[cit74] Tam N. M., Pham M. Q., Ha N. X., Nam P. C., Phung H. T. T. (2021). RSC Adv..

[cit75] Gimeno A., Mestres-Truyol J., Ojeda-Montes M. J., Macip G., Saldivar-Espinoza B., Cereto-Massagué A., Pujadas G., Garcia-Vallvé S. (2020). Int. J. Mol. Sci..

[cit76] Li G., De Clercq E. (2020). Nat. Rev. Drug Discovery.

[cit77] Gao J., Liang L., Zhu Y., Qiu S., Wang T., Zhang L. (2016). Int. J. Mol. Sci..

[cit78] Khan S. A., Zia K., Ashraf S., Uddin R., Ul-Haq Z. (2021). J. Biomol. Struct. Dyn..

[cit79] Wang J. (2020). J. Chem. Inf. Model..

[cit80] Koulgi S., Jani V., Uppuladinne M., Sonavane U., Nath A. K., Darbari H., Joshi R. (2020). J. Biomol. Struct. Dyn..

[cit81] Mody V., Ho J., Wills S., Mawri A., Lawson L., Ebert M. C. C. J. C., Fortin G. M., Rayalam S., Taval S. (2021). Commun. Biol..

[cit82] Chen H., Wei P., Huang C., Tan L., Liu Y., Lai L. (2006). J. Biol. Chem..

[cit83] Shekh S., Reddy K. K. A., Gowd K. H. (2021). J. Sulfur Chem..

[cit84] Raza S., Ranaghan K. E., van der Kamp M. W., Woods C. J., Mulholland A. J., Azam S. S. (2019). J. Comput.-Aided Mol. Des..

[cit85] Pettersen E. F., Goddard T. D., Huang C. C., Couch G. S., Greenblatt D. M., Meng E. C., Ferrin T. E. (2004). J. Comput. Chem..

[cit86] Harrison C. (2020). Nat. Biotechnol..

