# Supplementary material for: Drug repurposing and computational modeling for discovery of inhibitors of the main protease (Mpro) of SARS-CoV-2
Source: RSC Adv. 2021 Jul 2;11(38):23450–8. doi: 10.1039/d1ra03956c (PMC9036595; doi:10.1039/d1ra03956c)
Supplement: RA-011-D1RA03956C-s008 [file RA-011-D1RA03956C-s008.pdf]

Electronic Supplementary Material (ESI) for RSC Advances.
This journal is © The Royal Society of Chemistry 2021

Drug Repurposing and Computational Modeling for Discovering of Inhibitors of the Main Protease (M^{pro}) of SARS-CoV-2

José Rogério A. Silva, Hendrik G. Kruger and Fábio A. Molfetta

Supplementary Information

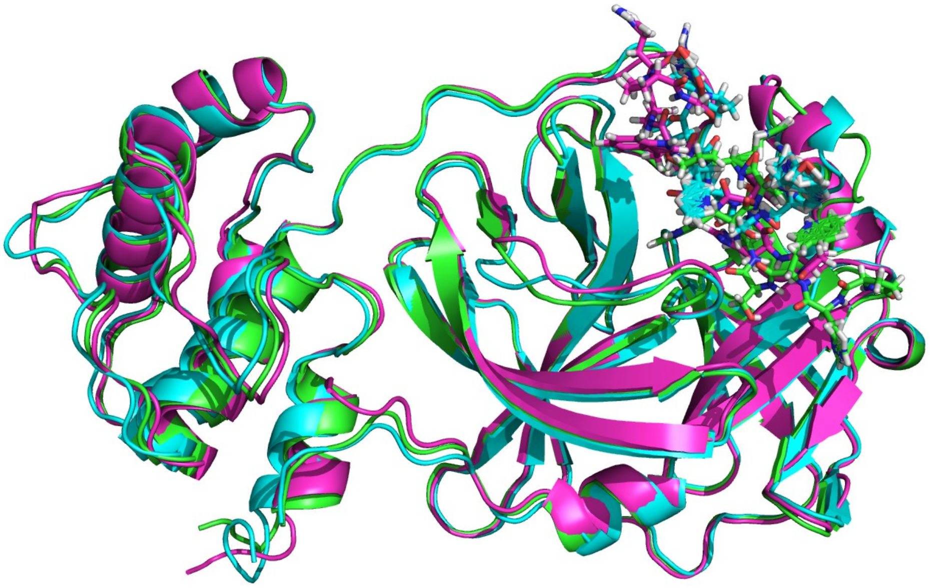

Fig. S1 Overlay of average structures of M^{pro} –triptorelin systems of 3 MD runs performed using random seed with different atomic velocities after 100 ns of MD simulations (RMSD = 0.76 ± 0.20 Å).

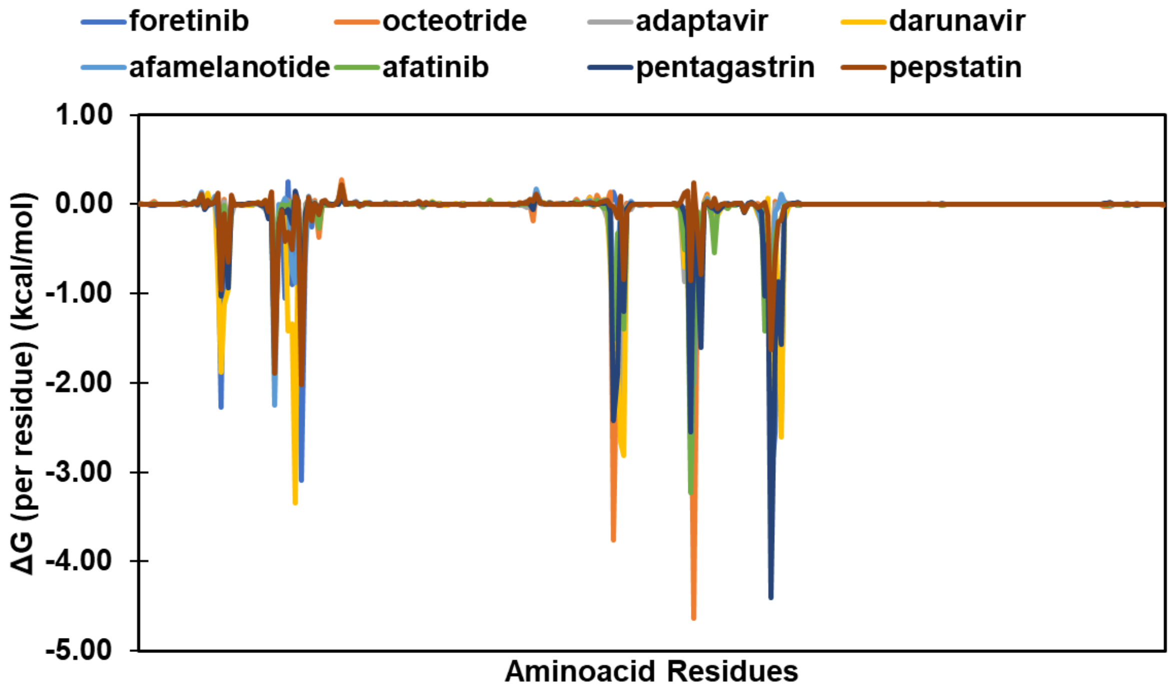

Fig. S2 Per-residue binding free energy decomposition (in kcal/mol) for other M^{pro}-drug systems.
